# Euler Force-Driven Siphon Valve Control for Precise Sequential Release in Centrifugal Microfluidic Chips

**DOI:** 10.3390/mi15101200

**Published:** 2024-09-27

**Authors:** Yu Lu, Hao Shen, Guangyao Chen, Kaichao Yang, Jing Zhang, Liwei Xue, Jianzhen Ou, Liguo Chen

**Affiliations:** 1 Jiangsu Provincial Key Laboratory of Advanced Robotics, School of Mechanical and Electrical Engineering, Soochow University, Suzhou 215123, China; 2 i-Lab, Suzhou Institute of Nano-Tech and Nano-Bionics (SINANO), Chinese Academy of Sciences (CAS), 398 Ruoshui Road, Suzhou 215123, China; 3 Key Laboratory of Advanced Technologies of Materials, Ministry of Education, School of Materials Science and Engineering, Southwest Jiaotong University, Chengdu 610031, China; jianzhen.ou@rmit.edu.au; 4 School of Engineering, RMIT University, Melbourne, VIC 3000, Australia

**Keywords:** controlled sequential release, Euler force, full release, angular acceleration

## Abstract

Controlling the fluids in centrifugal microfluidic chips for precise sequential release is critical for multi-step reactions and immunoassays. Currently, the traditional methods of liquid sequential release mainly rely on various types of microvalves, which face the problems of complex operation and high costs. Here, this work presents a method for driving liquid release using the Euler force. Under continuous acceleration and deceleration, the centrifugal and Euler forces can transfer the liquid from the sample chamber to the collection chamber. The liquid sequential release mechanism based on the Euler force was analyzed, which showed that the angular acceleration is key to the liquid release. Then, the geometrical parameters affecting the angular acceleration of complete release were investigated and simulated. Finally, based on the relationship between the geometrical parameters of the connecting channels and the angular acceleration of complete release, a simple and precise sequential release structure was designed, which allowed for a sequential and stable transfer of the liquid into the reaction chamber. The results showed that the proposed method is capable of transferring liquid, and its simple structure, low manufacturing cost, and ease of operation enable precise sequential liquid release in centrifugal microfluidic platforms.

## 1. Introduction

Centrifugal microfluidic platforms hold significant promise for applications in areas such as point-of-care testing (POCT), due to their operational simplicity, high level of integration, and high throughput [1]. By utilizing physical forces such as centrifugal force, the Coriolis force, and Euler force on centrifugal platforms, fluids can be precisely controlled to realize various functions such as transportation, metering, aliquoting, mixing, etc. [2,3], which provides great potential for integrating a variety of complicated functions into molecular diagnostic platforms, rendering them particularly suitable for POCT systems. However, controlling liquids to achieve precise sequential release has emerged as a critical challenge for biochemical assays such as enzyme-linked immunosorbent assays (ELISA) [4] and nucleic acid extraction [5].

Currently, it is common to use different types of valves in centrifugal microfluidic platforms for controlling the sequential release of fluids. The valves on centrifugal microfluidic chips are primarily categorized into active and passive valves. Active valves are usually reliable, because they are less reliant on machining accuracy and the stability of material properties. For example, representative active valves include electrowetting valves [6,7], diaphragm valves [8,9,10], and phase change microvalves [11,12,13]. Electrowetting valves can be remotely controlled with an external electric field to change the wetting of the valve, triggering its opening [14]. Diaphragm valves integrate a resilient diaphragm with a valve adaptor, allowing the valve to be reversibly opened and closed using a push rod [15]. Phase-change microvalves are typically single-use, requiring sacrificial materials to be pre-embedded in the chip prior to each use and utilizing temperature changes to open the valve [16]. Nevertheless, despite their robustness, active valves often necessitate more stringent external devices in addition to a control motor for state control, which increases system complexity and cost. To achieve simple, easy to manufacture and operate flow control, various types of passive valves have been developed. For example, hydrophobic valves [17,18], siphon valves [19,20,21], and capillary valves [22,23]. These passive valves do not require any external auxiliary devices besides the motor. They are primarily based on the capillary forces acting on centrifugal microfluidic chips and the centrifugal forces generated by the motor drive. However, they often involve additional complex processes, such as surface modification, and their stability tends to decrease over time, complicating the fabrication of centrifugal microfluidic chips. To avoid surface modification of the centrifugal microfluidic chip material, the valve can be opened by only controlling its motion state. Consequently, a method for decanting liquids using the Euler force has been proposed, which utilizes the Euler force as the driving force for liquid pumping to achieve the transfer of liquids. For example, Deng et al. designed a valve that enables the transfer of liquid, while the parameters affecting the valve turn-on were not studied in depth and cannot meet the needs of multi-step operations for liquids on centrifugal microfluidic chips [24]. Li et al. demonstrated multiple-unit operations of liquids on a chip; however, hydrophilic modification of the back-end channels was still required [25]. In addition, a dual-chamber structure utilizing the Euler force to decant liquids enabled sequential flow control [26,27,28]. However, the width of the outlet caused instability in liquid transfer and increased the risk of reagent contamination during multi-step sequential releases. Therefore, passive valves for controlling liquids in centrifugal microfluidic platforms to achieve precise sequential releases should be characterized by high stability, without the need for surface modification, and with a simple fabrication structure and control.

Here, we present a method for driving liquid release using the Euler force, without surface modification, and with a simple and stable structure. To achieve precise sequential liquid release in centrifugal microfluidic platforms, we performed an in-depth analysis and optimization of key structures. First, the liquid sequential release mechanism based on the Euler force was analyzed, which showed that the angular acceleration is key to the liquid release. Simulation experiments on an Euler-force-driven valve showed that the rotational speed range, inclination angle of the connecting channel, height, and width were the parameters that mainly affected the full release of the fluid. Subsequently, we conducted specific experiments on these parameters, which demonstrated that the minimum angular acceleration required for full fluid release was influenced by the rotational speed range, with a minimum observed at ±2000 rpm. The angular acceleration required for full fluid release increased with the increase in liquid surface tension and the height of the connecting channel, and decreased with increases in the inclination angle, width, and depth of the connecting channel. These trends remained consistent, even when the experiments were repeated with variations in other parameters. Finally, based on the relationship between the geometrical parameters of the connecting channels and the angular acceleration necessary for complete fluid release, a simple and precise sequential release structure was designed, allowing for sequential and stable transfer of the liquid into the reaction chamber. The results showed that the proposed method is capable of transferring liquid, and its simple structure, low manufacturing cost, and ease of operation enable precise sequential liquid release in centrifugal microfluidic platforms.

## 2. Materials and Methods

### 2.1. Materials

The centrifugal microfluidic chips were made of polymethylmethacrylate (PMMA). The structure on the chip was designed using AutoCAD 2018 software (Autodesk, CA, USA) and machined using a computerized numerical control (CNC) milling machine (JDHGT400T, Jiangsu Fanyang Electronics Co., Ltd. Changzhou, China). The microstructures on the centrifugal chip were sealed using a pressure-sensitive film (T7120N-10F50, Zhejiang Ouren New Material Co., Ltd. Jiaxing, China). The liquid used in this experiment was made by adding food coloring to deionized water. Specifically, three different liquids were prepared by adding different amounts of pigment to a fixed volume of deionized water. The three liquids were used to measure their contact angles on the surface of the same connecting channel.

### 2.2. Equipment Development

The centrifugal microfluidic control system was composed of a servo motor (MS1H4-20B3, Huichuan Technology, Suzhou, China), a driver (SV630NS1R6, Huichuan Technology, Suzhou, China), and a programmable logic controller (PLC, Easy501-08, Huichuan Technology, Suzhou, China). The rotation of the motor was controlled by a computer program written in AutoShop V4.10.0.0 (Huichuan Technology, Suzhou, China). A high-speed camera (SH6-109-M-160, Shenzhen Shenshi Intelligent Technology Co., Ltd. Shenzhen, China) was used to capture images of the liquid flow on the centrifugal microfluidic chip during high-speed rotation, and a continuous light source (EF-220, Jinbei, Shanghai, China) was installed for illumination.

## 3. Results and Discussion

### 3.1. Design and Working Principle

**Figure 1:**
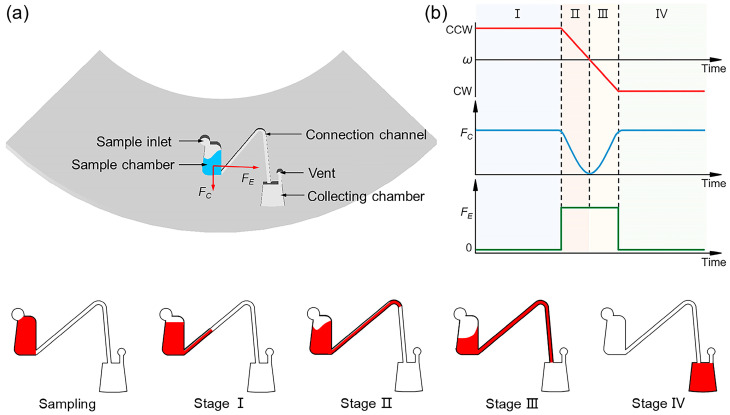
Schematic diagram of the structure and working principle of the Euler-force-driven valve on a centrifugal microfluidic chip. (**a**) Structure of the Euler-force-driven valve. (**b**) Processes of rotational speed (*ω*), centrifugal force (*F_C_*), and Euler force (*F_E_*) during fluid transfer, along with the states of different liquids in the four stages.

Figure 1a illustrates the primary structural design of the presented Euler-force-driven valve, including the sample inlet, sample chamber, connection channel, collection chamber, and vent hole. When a centrifugal microfluidic chip rotates, the fluid within the chip is primarily subjected to centrifugal force (*F_C_*) and the Euler force (*F_E_*). The centrifugal force is generated when the fluid moves within a rotating frame of reference, and the Euler force arises when the chip accelerates or decelerates. These forces are described by Equations (1) and (2) [29] and can be controlled by the angular velocity *ω*, as shown in Figure 1a.

(1) $$F_{C}=\rho \omega^{2}r$$

(2) $$F_{E}=\rho r\frac{d\omega }{\mathrm{dt}}$$

where *ρ* is the mass density of the fluid and *r* is the distance of the fluid from the center axis.

In achieving liquid release, the centrifugal platform provides an Euler force much larger than the centrifugal force, to drive the liquid transfer from the sample chamber along the connecting channel to the collection chamber, as shown in Figure 1. The movement of the liquid in the sample chamber is affected by both centrifugal and Euler forces. The centrifugal force moves the liquid in the direction of the radius, while the Euler force drives the liquid in the direction perpendicular to the centrifugal force. To facilitate the liquid transfer from the sample chamber to the collection chamber, the centrifugal microfluidic chip first rapidly decelerates in a counterclockwise (CCW) direction and then accelerates clockwise (CW). The relationship between *ω*, *F_C_*, and *F_E_* during release is shown in Figure 1b. Deionized water with red pigment was first added to the sample chamber, and the process for achieving liquid transfer was divided into four stages. In Stage I, when the centrifugal microfluidic chip was rotated at a constant rotational speed in a CCW direction, the liquid stayed at the bottom of the sample chamber due to the centrifugal force. In Stage II, through rapid deceleration, the Euler force drove the fluid in a CCW direction, causing the fluid to rise along the connecting channel until the rotational speed reached zero. At this point, the centrifugal force was minimized, and the liquid reached the highest point of the channel, where the two segments join. In Stage III, the chip was accelerated rapidly in a CW direction. At the beginning of rotation, the Euler force dominated due to being greater than the centrifugal force. The Euler force continued to drive the liquid to move in a CCW direction. As the rotational speed gradually increased, the centrifugal force dominated over the Euler force, causing the liquid to move radially until it filled the entire channel. In Stage IV, when the chip rotated CW at a constant rotational speed, the centrifugal force fully actuated the liquid, causing it to be transferred from the sample chamber to the collection chamber.

### 3.2. Design of Geometric Parameters of the Connecting Channel

**Figure 2:**
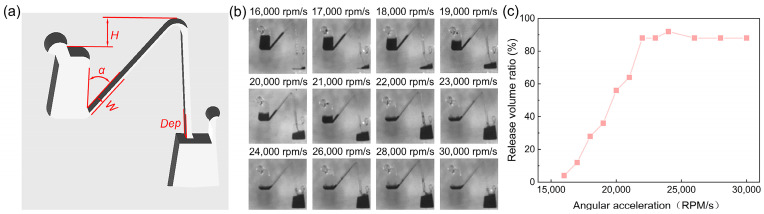
Schematic diagram of geometric parameters. (**a**) Schematic representation of the geometric parameters of the valve structure. (**b**) Plot of the liquid levels in the sample chamber compared to those in the collection chamber at various angular accelerations. (**c**) Correlation between angular acceleration and the release volume ratio.

In order to achieve precise control over the sequential release of liquids from multiple Euler-force-driven valves, this work explored the effect of various geometric factors on the angular acceleration of complete release. The specific geometrical parameters explored with the Euler-force-driven valve included the height of the connecting channel *H*, the inclination angle α, the depth of the channel *Dep*, and the width of the channel *W* (Figure 2a). First, the magnitude of the angular acceleration plays an important role in the liquid transfer process. If the angular acceleration is too small, it cannot provide enough actuation force for the liquid; if the angular acceleration is too large, this may affect the proper layout of multiple actuated valves. To control the sequential release of the liquid, it is important to determine the full release angular acceleration, which is defined as the minimum angular acceleration required to achieve the maximum volume ratio. The release volume ratio is defined as the ratio of the volume of liquid in the collection chamber to the original volume in the sample chamber (Figure 2b). In a structure with a height of 2 mm, an inclination angle of 40°, a depth of 2 mm, and a width of 0.5 mm, 25 μL of deionized water with pigment was added to the sample chamber. The chip was then rotated in a counterclockwise direction at an initial rotational speed of 3000 rpm. The liquid was transferred by decelerating in the counterclockwise direction and accelerating in the clockwise direction with different angular accelerations, and finally rotated in the clockwise direction at a final rotational speed of 3000 rpm. The results showed that the release volume ratio increased with the angular acceleration. However, the release volume ratio reached a stabilized value when the angular acceleration exceeded 22,000 rpm/s (Figure 2c). At this point, 22,000 rpm/s was referred to as the full release angular acceleration.

### 3.3. COMSOL Simulation

In order to verify the feasibility of the experiments, the parameters were analyzed through COMSOL Multiphysics 6.0 numerical simulation prior to the specific experiments. A two-phase flow phase field approach was employed to track the interface between two immiscible fluids. This can be expressed in the following equations and solved with the Navier–Stokes (Equation (3)) and the continuity (Equation (4)).

(3) $$\rho \frac{\partial u}{\partial t}+\rho \left(u\cdot \nabla \right)u=\nabla \cdot \left[-\mathrm{pI}+K\right]+F$$

(4) ρ∇·u=0

where *ρ* is the fluid density, *t* is time, *u* is the fluidic velocity vector, *p* is the pressure, *I* is the unitary tensor, and *F* is the volume force acting on the fluid.

In the phase–field interface, the dynamics of two-phase flow are governed by the Cahn–Hilliard equation. This equation is used to track the diffusion interface, which is the region between two immiscible liquids where the dimensionless phase field variable φ transitions from −1 to 1. The propagation of the two-phase flow interface and the initialization of the phase field are controlled by Equation (5):

(5) $$\frac{\partial \varphi }{\partial t}+u\cdot \nabla \varphi =\nabla \cdot \frac{\gamma \lambda }{\varepsilon^{2}}\nabla \Psi$$

where *γ* is the mobility, *λ* is the mixing energy density, *ε* is the interface thickness parameter, and *Ψ* is referred to as the phase field covariate.

**Figure 3:**
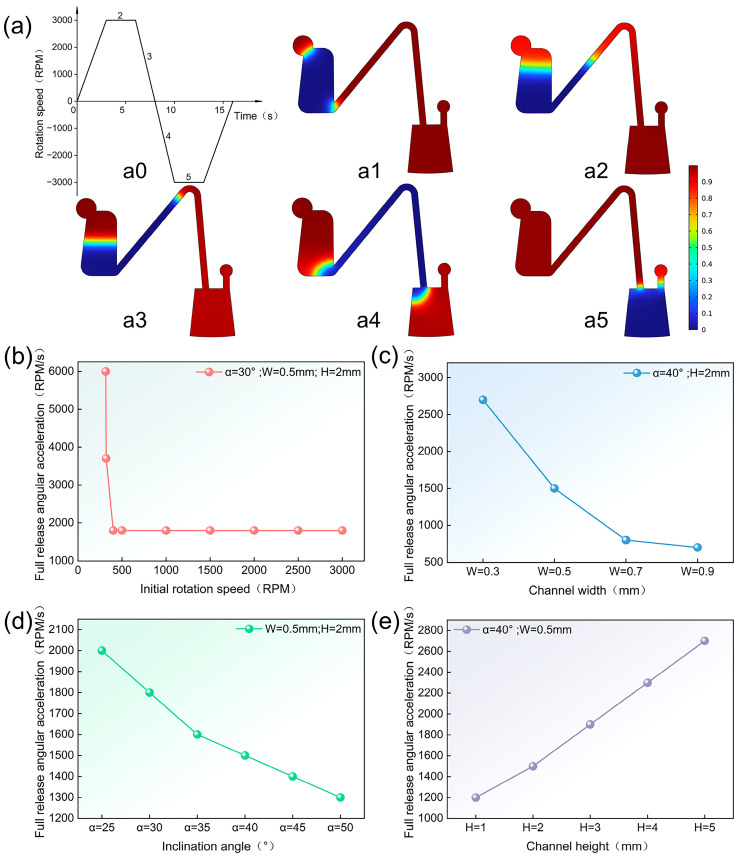
Simulation results of Euler-force-driven fluid transfer. (**a**) Schematic simulation of the liquid transfer process (**a0**) denotes the rotation speed versus time during simulation, (**a1**–**a5**) illustrate the simulation process of liquid transfer, with the gas phase depicted in red and the aqueous phase in blue. (**b**) Simulation results of initial rotational speed and full release angular acceleration. (**c**) Simulation results of connecting channel width and full release angular acceleration. (**d**) Simulation results of connecting channel inclination angle and full release angular acceleration. (**e**) Simulation results of connecting channel heights and full release angular acceleration.

A two-dimensional, two-phase laminar flow model was utilized, with water and air as the fluids, having densities of 1000 kg/m^3^ and 1.61 kg/m^3^, respectively, and dynamic viscosities of 8.94 × 10^−4^ Pa·s and 1.86 × 10^−5^ Pa·s at a temperature of 25 °C. The rotational domain in the dynamic mesh was added, and an interpolation function was called to represent the change in rotational speed of the valve structure over time. The centrifugal and Euler forces generated by the rotation of the valve structure around the midpoint of rotation were utilized to represent the volumetric forces. Zero-pressure boundary conditions were used for both the outlet and inlet boundaries, while no-slip boundary conditions were implemented for all other boundaries. The contact angle of the wetted wall was 93.4°. The numerical simulation results obtained are shown in Figure 3. The results indicate that the full release acceleration decreased with an increased in the initial rotational speed, and the full release angular acceleration no longer changed after reaching a certain initial rotational speed (Figure 3b). For the same rotational speed range, the full release angular acceleration decreased with increasing connection channel width and inclination angle (Figure 3c,e), and increased with increasing connection channel height (Figure 3d). Thus, the necessity of the parameters proposed for the study was verified, while the trends among the rotational speed range, inclination angle of the connecting channel, width, height, and the full release angular acceleration, as indicated by the simulation results, provided a preliminary reference for the subsequent experiments.

### 3.4. Study of Parameters Affecting Controlled Sequential Release

**Figure 4:**
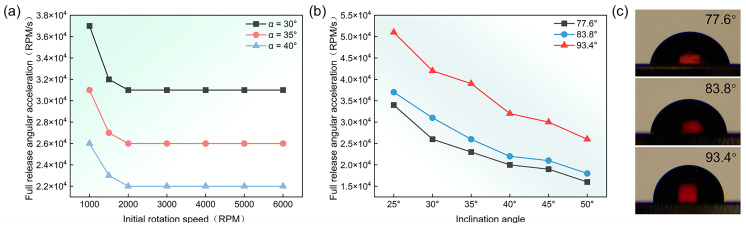
Study of rotational speed range and liquid surface tension. (**a**) Trends in full release angular acceleration across different rotational speed ranges. (**b**) The relationship between liquid surface tension and full release angular acceleration. (**c**) Contact angles of different liquids.

In order to combine the use of Euler-force-driven valves that can be triggered by different full release angular accelerations, we aimed to enable the controlled sequential release of fluids on centrifugal microfluidic platforms. Consequently, we investigated the effects of the rotational speed range, liquid surface tension, and the geometrical parameters of the connecting channel on the full release angular acceleration. First, the rotational speed range was defined as the starting rotational speed of the centrifugal microfluidic chip in the counterclockwise direction to the terminating rotational speed in the clockwise direction. As shown in Figure 4a, the full release angular acceleration decreased with increasing rotational speed, until it reached a minimum point at a rotational speed range of ±2000 rpm or more, when all other parameters remained constant. This was due to the fact that, in the high rotational speed range, the centrifugal force generated was much greater than the Euler force, which prevented the Euler force from driving the fluid. However, it was advantageous to use a wider rotational speed range to increase the time for the Euler force effect. Nevertheless, the full release angular acceleration did not change when the rotational speed range was increased from ±2000 rpm to ±6000 rpm, as shown in Figure 4a, because the Euler force on the fluid was counteracted by the centrifugal force generated at high rotational speeds. The effective rotational speed range may vary with the geometric parameters of the connection channel. Therefore, three different sets of inclination angles were used to investigate the relationship between the rotational speed range and full release angular acceleration. From the experimental results obtained, it was clear that the effective rotational speed range was essentially unchanged with variations in the geometrical parameters of the connecting channel. Consequently, the rotational speed range chosen for this experiment was ± 3000 rpm.

To investigate the relationship between the surface tension of a liquid and its full release angular acceleration, we used three liquids with different formulations to measure their contact angles on the surface of the same connecting channel. The contact angles were measured to be 77.6°, 83.8°, and 93.4° by placing 1 μL droplets on a contact angle meter (Figure 3c).

The relationship between the contact angle *θ* of a droplet and the surface tension can be expressed by Young’s equation as [30]

(6) $$\gamma =\frac{\gamma_{sv}-\gamma_{sl}}{cos\theta }$$

where *γ* is the liquid–gas interfacial tension, *γ_sv_* is the solid–gas interfacial tension, and *γ_sl_* is the solid–liquid interfacial tension.

From Equation (6), it can be seen that the liquid surface tension increases with an increasing contact angle. Figure 4b illustrates that, for the same inclination angle, the full release angular acceleration increased with an increasing liquid surface tension. Even when the tests were conducted using different structures, the conclusions obtained remain consistent.

**Figure 5:**
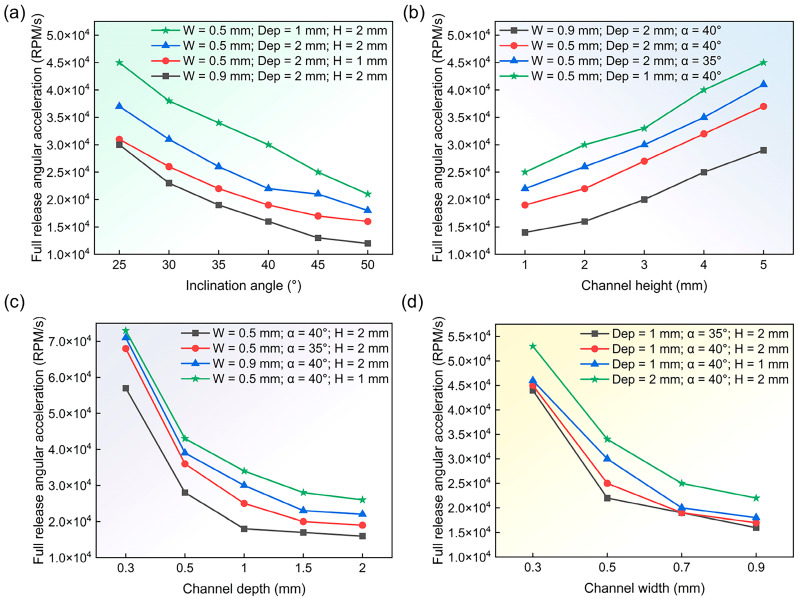
Study of the geometric parameters of the connecting channel. (**a**) The relationship between the inclination angle of the connecting channel and the angular acceleration upon complete release. (**b**) The relationship between the height of the connecting channel and the angular acceleration upon complete release. (**c**) Trends in full release angular acceleration for different depths of the connecting channel. (**d**) Trends in full release angular acceleration for different widths of the connecting channel.

Second, four distinct geometrical parameters (inclination angle, height, depth, and width of the connecting channel) were selected to investigate their effect on the full release angular acceleration (Figure 5). The rotational speed range employed in these experiments was ±3000 rpm, the contact angle of the liquid was 83.8°, and each set of experiments was validated using four different sets of parameters, with the aim of ensuring that the conclusions drawn were generalizable and consistent. Figure 5a shows that for the same liquid surface tension, width, depth, and height of the connecting channel, the full release angular acceleration decreased with increasing inclination angle of the connecting channel. Figure 5b illustrates that, for the same width, depth, and inclination angle of the connecting channel, the full release angular acceleration increased with the height of the connecting channel. Similarly, the full release angular acceleration decreased with the increasing depth and width of the connecting channel, with other factors being equal (Figure 5c,d). The above findings also show that the full release angular acceleration can be flexibly varied over a wide range by adjusting the geometric parameters of the connecting channel. Moreover, based on these conclusions, precise and controlled sequential release of liquids on centrifugal microfluidic platforms can be effectively achieved through the integration of Euler-force-driven valves, which are activated by varying full release accelerations.

### 3.5. Design and Implementation of Sequential Release Structures

**Figure 6:**
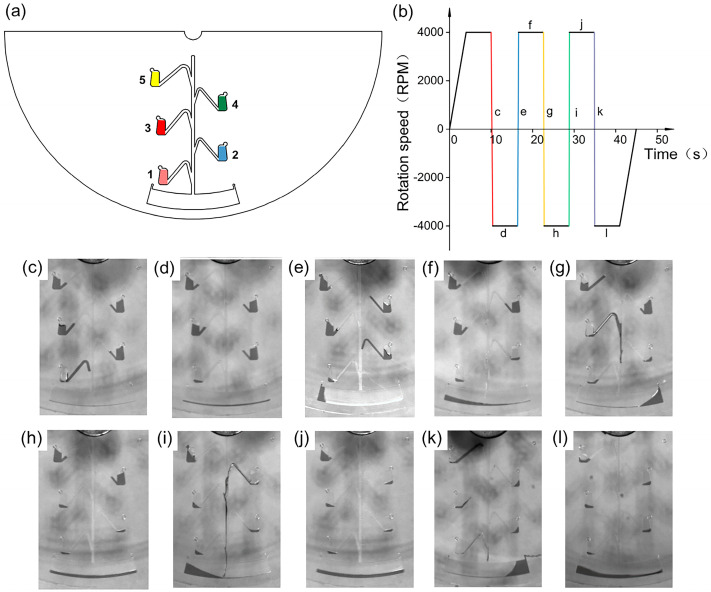
Schematic diagram of controlled sequential release. (**a**) Design drawings of five Euler-force-driven valve sequential release configurations. (**b**) Plot of speed versus time during sequential release. Within the ±4000 RPM rotation speed range, the running angular accelerations are 20,000, 35,000, 50,000, 95,000, and 100,000 RPM/s from left to right. (**c**–**l**) Physical images corresponding to (**b**) during sequential release.

Controlling the fluid to achieve a precise sequential release is one of the most complex steps in centrifugal microfluidic platforms. For instance, isothermal amplification processes typically include operations such as sample processing, nucleic acid extraction, and amplification reactions, and therefore require controlled and precise release of different reagents in a sequential manner [31]. Here, based on the above design principle and the relationship between each parameter and the full release angular acceleration, a simple and precise sequential release structure was designed, as shown in Figure 6a. Since the Euler-force-driven valves are located in different radial positions, the turning on of an Euler-force-driven valve is mainly related to the Euler force. Equation (2) shows that as Euler-force-driven valves get closer to the center of rotation, the smaller their centrifugal radius becomes and the greater the minimum acceleration required for the complete release of the fluid. Therefore, to achieve sequential release of the fluid, the height, inclination angle, depth, and width of each connecting channel were appropriately adjusted so that each of the Euler-force-driven valves had a sequentially increasing full release angular acceleration. The geometric parameters of the designed sequential release structure are listed in Table 1. According to the control of the rotational speed (Figure 6b), the centrifugal microfluidic chip initially rotates counterclockwise at 4000 rpm, then decelerates to 0 with an angular acceleration of 20,000 rpm/s to activate valve 1 (Figure 6c), and immediately accelerates clockwise to 4000 rpm with an angular acceleration of 20,000 rpm/s, subsequently rotating clockwise at a constant speed of 4000 rpm to realize the release of valve 1 (Figure 6d). Subsequently, valve 2 is activated by decelerating to 0 with an angular acceleration of 35,000 rpm/s (Figure 6e), then immediately accelerating counterclockwise to 4000 rpm with an angular acceleration of 35,000 rpm/s, and finally rotating counterclockwise at a constant speed of 4000 rpm to enable the release of valve 2 (Figure 6f). By running counterclockwise and clockwise at ±4000 rpm with angular accelerations of 50,000, 95,000, and 100,000 rpm/s, the liquids in valves 3, 4, and 5 can be transferred sequentially into the reaction chamber (Figure 6g–l). Liquid sequential release videos are provided in the Supplementary Information.

## 4. Conclusions

In this work, a method for driving liquid release using Euler force was presented. First, the liquid sequential release mechanism based on the Euler force was analyzed, which deduced that the angular acceleration is the key to the liquid release. The minimum angular acceleration required for complete fluid release is associated with the rotational speed. This angular acceleration is minimized when the rotational speed is ±2000 rpm. Furthermore, the angular acceleration necessary for complete liquid release increases with the surface tension of the liquid. Additionally, the geometric parameters of the connection channel in an Euler-force-driven valve influence the fluid’s full release acceleration. Specifically, the angular acceleration required for complete liquid release decreases with the increasing inclination angle, width, and depth of the connection channel, while it increases with the height of the connection channel. Finally, based on the relationship between the geometrical parameters of the connecting channels and the angular acceleration of complete release, a simple and precise sequential release structure was designed, which allows for a sequential and stable transfer of the liquid into the reaction chamber. Our Euler-force-driven sequential liquid release structure enables liquid transfer, without requiring tedious steps such as surface modification of the chip material, and is simple, stable, and reliable, without the need for complex external equipment. We strongly believe that it can be used for immunoassays or clinical tests, such as ELISA and nucleic acid extraction.

## Figures and Tables

**Table 1:** The geometric parameters of the controlled sequential release structures.

Structure	A (°)	H (mm)	W (mm)	Dep (mm)
1	40	2.4	0.9	2
2	40	2.2	0.7	1.5
3	40	3	0.9	2
4	40	2.2	0.7	2
5	50	1.9	0.9	1.5

## Data Availability

The experimental data are available from the authors.

## Supplementary Materials

The following supporting information can be downloaded at: https://www.mdpi.com/article/10.3390/mi15101200/s1, Video S1: Liquid sequence release video.

## References

[1] Li S., Zhang Y., Liu J., Wang X., Qian C., Wang J., Wu L., Dai C., Yuan H., Wan C. (2024). Fully integrated and high-throughput microfluidic system for multiplexed point-of-care testing. Small.

[2] Zong N., Gao Y., Chen Y., Luo X., Jiang X. (2022). Automated centrifugal microfluidic chip integrating pretreatment and molecular diagnosis for hepatitis b virus genotyping from whole blood. Anal. Chem..

[3] Wang S., Qi W., Wu S., Yuan J., Duan H., Li Y., Lin J. (2022). An automatic centrifugal system for rapid detection of bacteria based on immunomagnetic separation and recombinase aided amplification. Lab Chip.

[4] Lee B.S., Lee J.N., Park J.M., Lee J.G., Kim S., Cho Y.K., Ko C. (2009). A fully automated immunoassay from whole blood on a disc. Lab Chip.

[5] Zhao X., Huang Y., Li X., Yang W., Lv Y., Sun W., Huang J., Mi S. (2022). Full integration of nucleic acid extraction and detection into a centrifugal microfluidic chip employing chitosan-modified microspheres. Talanta.

[6] Biswas G.C., Watanabe T., Carlen E.T., Yokokawa M., Suzuki H. (2016). Switchable hydrophobic valve for controlled microfluidic processing. Chemphyschem.

[7] Guo Z., Jiao Y., Wang H., Zhang C., Liang F., Liu J., Yu H., Li C., Zhu G., Wang Z. (2019). Self-powered electrowetting valve for instantaneous and simultaneous actuation of paper-based microfluidic assays. Adv. Funct. Mater..

[8] Cai Z., Xiang J., Wang W. (2015). A pinch-valve for centrifugal microfluidic platforms and its application in sequential valving operation and plasma extraction. Sens. Actuator B-Chem..

[9] Kim T.H., Sunkara V., Park J., Kim C.J., Woo H.K., Cho Y.K. (2016). A lab-on-a-disc with reversible and thermally stable diaphragm valves. Lab Chip.

[10] Chen Y., Shen M., Zhu Y., Xu Y. (2019). A novel electromagnet-triggered pillar valve and its application in immunoassay on a centrifugal platform. Lab Chip.

[11] Cho Y.K., Lee J.G., Park J.M., Lee B.S., Lee Y., Ko C. (2007). One-step pathogen specific DNA extraction from whole blood on a centrifugal microfluidic device. Lab Chip.

[12] Amasia M., Cozzens M., Madou M.J. (2012). Centrifugal microfluidic platform for rapid PCR amplification using integrated thermoelectric heating and ice-valving. Sens. Actuator B-Chem..

[13] Park J.M., Cho Y.K., Lee B.S., Lee J.G., Ko C. (2007). Multifunctional microvalves control by optical illumination on nanoheaters and its application in centrifugal microfluidic devices. Lab Chip.

[14] Xia Y., Song C., Meng Y., Xue P., de Mello A.J., Gao Q., Stavrakis S., Ma S., Cao X. (2022). An addressable electrowetting valve for centrifugal microfluidics. Sens. Actuator B-Chem..

[15] Qian C., Wan C., Li S., Xiao Y., Yuan H., Gao S., Wu L., Zhou M., Feng X., Li Y. (2023). On-line dual-active valves based centrifugal microfluidic chip for fully automated point-of-care immunoassay. Anal. Chem..

[16] Sunkara V., Kumar S., Del Río J.S., Kim I., Cho Y.K. (2021). Lab-on-a-disc for point-of-care infection diagnostics. Acc. Chem. Res..

[17] Ishida T., McLaughlin D., Tanaka Y., Omata T. (2018). First-come-first-store microfluidic device of droplets using hydrophobic passive microvalves. Sens. Actuator B-Chem..

[18] Dal Dosso F., Tripodi L., Spasic D., Kokalj T., Lammertyn J. (2019). Innovative hydrophobic valve allows complex liquid manipulations in a self-powered channel-based microfluidic device. ACS Sens..

[19] Siegrist J., Gorkin R., Clime L., Roy E., Peytavi R., Kido H., Bergeron M., Veres T., Madou M. (2010). Serial siphon valving for centrifugal microfluidic platforms. Microfluid. Nanofluidics.

[20] Zehnle S., Schwemmer F., Bergmann R., von Stetten F., Zengerle R., Paust N. (2015). Pneumatic siphon valving and switching in centrifugal microfluidics controlled by rotational frequency or rotational acceleration. Microfluid. Nanofluidics.

[21] Zhu Y., Chen Y., Xu Y. (2018). Interruptible siphon valving for centrifugal microfluidic platforms. Sens. Actuator B-Chem..

[22] Choi J., Kang D., Han S., Kim S.B., Rogers J.A. (2017). Thin, soft, skin-mounted microfluidic networks with capillary bursting valves for chrono-sampling of sweat. Adv. Healthc. Mater..

[23] Chen J., Huang P., Lin M. (2008). Analysis and experiment of capillary valves for microfluidics on a rotating disk. Microfluid. Nanofluidics.

[24] Deng Y., Fan J., Zhou S., Zhou T., Wu J., Li Y., Liu Z., Xuan M., Wu Y. (2014). Euler force actuation mechanism for siphon valving in compact disk-like microfluidic chips. Biomicrofluidics.

[25] Li N., Shen M., Zhu Y., Xu Y. (2022). Euler force-assisted sequential liquid release on the centrifugal microfluidic platform. Sens. Actuator B-Chem..

[26] Shih C., Cheng Y., Wu H., Chang C., Zhao Y. (2022). Sequential flow control by liquid decanting on a centrifugal platform. Sens. Actuator A-Phys..

[27] Fakhari S., Pishbin E., Navibakhsh M., Maghazeh M., Eghbal M. (2019). Implementing series of dual-chamber units for sequential loading of the liquids in centrifugal microfluidic platforms. Microfluid. Nanofluidics.

[28] Shih C., Chen J., Zhao Y. (2020). Automated Protein Purification on a Centrifugal Platform. ECS J. Solid. State Sci. Technol..

[29] He X., Wang X., Ge C., Li S., Wang L., Xu Y. (2022). Detection of VEGF165 in whole blood by differential pulse voltammetry based on a centrifugal microfluidic chip. ACS Sens..

[30] Sedev R. (2011). Surface tension, interfacial tension and contact angles of ionic liquids. Curr. Opin. Colloid. Interface Sci..

[31] Li Z., Xu X., Wang D., Jiang X. (2023). Recent advancements in nucleic acid detection with microfluidic chip for molecular diagnostics. Trac-Trends Anal. Chem..

